# Trends in Adult Cochlear Implant Access and Uptake Across Ten Years of Reported Data

**DOI:** 10.3390/audiolres16010019

**Published:** 2026-01-29

**Authors:** Patrick D’Haese, Paul Van de Heyning, Javier Gavilan, Mario Emilio Zernotti, Paula Greenham

**Affiliations:** 1 MED-EL GmbH, 6020 Innsbruck, Austria; 2 Faculty of Medicine and Pharmacy, Vrije Universiteit Brussel, 1050 Brussel, Belgium; 3 Hearing Health Forum EU, 6020 Innsbruck, Austria; paul@vandeheyning.com; 4 Department of Otorhinolaryngology, Head and Neck Surgery, Antwerp University Hospital, University of Antwerp, 2000 Antwerp, Belgium; 5 Department of Translational Neurosciences, Faculty of Medicine and Health Sciences, University of Antwerp, 2000 Antwerp, Belgium; 6 Department of Otorhinolaryngology, La Paz University Hospital, Castellana 261, 28046 Madrid, Spain; javier.gavilan@salud.madrid.org; 7 Department of Otorhinolaryngology, Sanatorio Allende, Córdoba 5000, Argentina; mario.zernotti@gmail.com; 8 Greenham Research Consulting Ltd., Swindon SN6 8LP, UK; paulagreenham@gmail.com

**Keywords:** uptake, hearing loss, penetration, prevalence, cochlear implant

## Abstract

Background: Adults with severe to profound hearing loss have limited access to cochlear implants (CIs). The objective of this study was to assess the evidence to establish whether the uptake rate of CIs has changed over the past decade. Methods: A PubMed search, supplemented with manual searching, identified 15 relevant papers published from 2000 to 4 February 2025 reporting the uptake rate of CIs in adults. In addition, new calculations of uptake rates were made for 2019, based on total numbers of CIs implanted and the prevalence of hearing loss from the 2019 Global Burden of Disease Study. Results: There was a lack of published data on the uptake rates for cochlear implants, with very little consensus in the methods used across studies. The overall uptake rates for adults and children combined, calculated for 2019 using the Lancet Global Burden of Disease Study, showed that uptake is still ≤20% for those with profound to complete hearing loss in most high-income countries. When the global population is considered (including high- to low-income countries), it is merely 2.5%. Conclusions: Despite the cochlear implant awareness activities of recent years, the percentage of profoundly deaf individuals with cochlear implants, even in high-income countries, remains low. Uptake rates are much worse than those for hearing aid use for severe to profound deafness. Better and more accurate data must be gathered on the number of CI recipients to meet the reporting requirements of the World Health Organisation’s report on hearing.

## 1. Introduction

Cochlear implantation is an established, effective procedure for individuals with severe to profound hearing loss [1]. It leads to improved hearing performance, better quality of life, more independence, reduced hearing disability and less loneliness [2]. There is also emerging evidence that maintaining good hearing plays an important role in preserving cognitive function as we age [3,4,5]. The percentage of adults who meet the criteria for implantation and proceed to have surgery is generally referred to as the uptake rate. Considering the substantial proven benefits of implantation, it is surprising that the uptake rate in adults is limited [6,7,8]. In 2016 Sorkin and Buchman reported in depth on the access to cochlear implants (CIs) in six developed countries. They found that, irrespective of the differing funding models for different countries, adult uptake was less than 10%. Equally low adult uptake was observed in both insurance-based systems and government-funded health care systems. This was in marked contrast to paediatric uptake rates, which approached 100% in some countries [7]. The reasons given for this were multi-faceted and encompassed societal attitudes, restricted funding, fear of surgery and a lack of professional knowledge about referral criteria [9,10]. Anecdotal evidence suggests that there has been little change in this situation over the past decade, despite efforts to address the issue. The availability of CIs is even poorer in low- and middle-income countries, where around 80% of the hearing-impaired population live [11,12]. Here access to basic hearing services is limited, let alone essential technologies such as CIs. Funding for CI surgery is often non-existent, and individuals must rely on charitable funding or personal wealth. The World Report on Hearing provides clear guidance for both policy makers and clinicians with the aim of improving hearing care worldwide, including access to advanced hearing aid and implantable hearing device technologies [13,14]. A good understanding of CI uptake data is critical in achieving this as it contributes to health planning, equity monitoring and policy benchmarking.

The recognition of the issue of the limited access to CIs faced by adults with severe to profound hearing loss has resulted in an increase in awareness activities. The aim of these was to raise awareness of CIs not just in the general public but also amongst hearing professionals [15,16,17,18]. Audiologists and Ear, Nose and Throat specialists often have limited knowledge of CI criteria and do not refer their patients for further assessment [17]. The type of awareness activities undertaken are well-described in Kang et al. (2024) [15]. They ranged from advocacy via patient groups or individuals to digital and print media campaigns. Measuring the success of these activities in improving access, however, is difficult. Analysis of Google Trends data showed that a Cochlear Implant Awareness Day improved Google search rates for CIs the week after the event, but this improvement was not sustained in the longer term [15]. Likewise, a digital awareness campaign run in mainstream newspapers and specialist magazines had no effect on long-term awareness [18]. Raine et al. (2016) reported on a training scheme targeted at referring audiologists to their centre [10]. After the training, there was an increase in knowledge of candidacy criteria and a threefold increase in referrals. The key question remains, however, of whether awareness activities have had any effect on the adult uptake rates in the longer term.

A European report gathered data either directly from clinics or from publicly available databases and assessed if there were any changes in CI uptake between 2010 and 2016 [6]. Rates of implantation per million of the population were compared between 2010 and 2016 for the countries where data was available. There was little growth in adult implantation rates between 2010 and 2016, except in Belgium, Switzerland, Finland and the UK [6]. Another paper reports an increase in adult uptake rates in Australia over the period from 2000 to 2015 but reports a plateau in numbers from 2015 onwards [19]. This growth is initially attributed to an increase in public and clinical awareness of CIs, but latterly they comment that the number of CIs is not keeping up with demand due to limited funding and clinical capacity. Evidence was also provided that the largest growth in CI rates was in the over-75 s [19]. This indicates that CI rates in this previously underserved population may finally be catching up to those in the 50–64 age ranges [7].

The objective of this study was to assess the change in uptake rates of CIs over the past decade. The study had two parts: First the evidence of a change in uptake over time reported in the literature was assessed. Then, new calculations were made of current uptake rates based on the Global Burden of Disease Study 2019 [20].

## 2. Methods

### 2.1. Part 1

**Figure 1:**
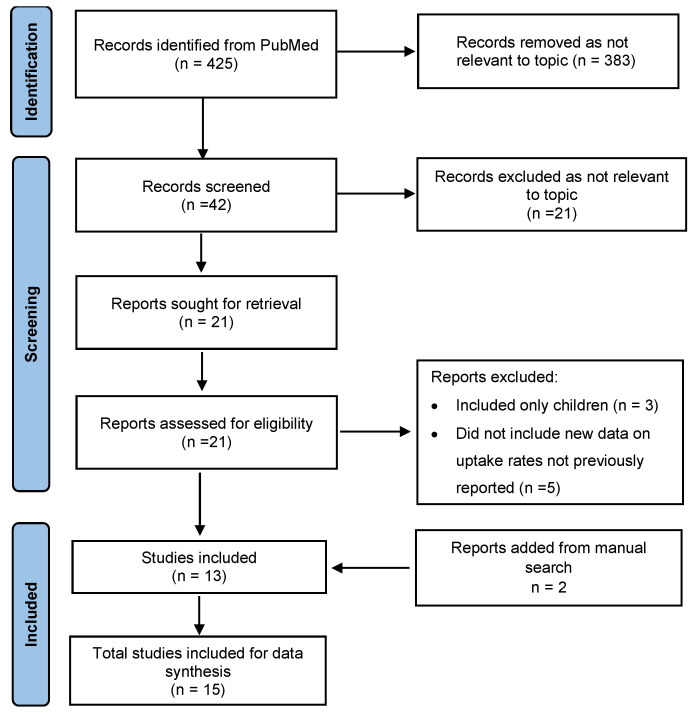
Prisma flow for PubMed literature review.

In the first phase, a literature review was conducted via PubMed to identify papers including the search terms “cochlear implant” and “uptake”, “penetration”, “prevalence” or “utilization/utilisation”. Papers were included that reported the CI “uptake rate”, defined in this paper as the number of adults or children receiving a CI as a proportion of either the total population that would meet the criteria for a CI or the total population. Other studies sometimes refer to this as implant utilisation or market penetration. Papers were included that were in English from 2000 to 4 February 2025. Out of a total of 425 hits, 42 abstracts were selected for further screening. After review, 21 relevant papers were identified for full-text review. Papers were excluded if reporting data only on children or if the data on uptake rates had already been reported in a previous paper. Data was extracted from 13 papers to provide information on a trend in CI uptake rates over time. Two additional papers were added after manual searching (Figure 1).

### 2.2. Part 2

To calculate the new uptake rates, estimates of the total cases of profound or complete hearing loss per country were extracted from the Global Burden of Disease 2019 report, which represents the estimated number of individuals with hearing loss in that country at a specific time point. The total number of cochlear implantations carried out by 2019 in a range of countries was established based on internet searches and prior knowledge. This data was not readily available in many countries and was gathered from a variety of sources. Where data on the number of implants was only available for the current year, the value for 2019 was interpolated based on the reported annual CI implantation rate. Uptake was defined as either the number of CI recipients or the number of CI devices implanted as a proportion of the number of potential candidates.

It was decided to only include profound or complete hearing loss as these individuals would comfortably meet the implant criteria across all countries. All individuals with complete or profound hearing loss were considered as potential candidates for the purposes of this analysis. It was also decided not to use the 2021 Global Burden of Disease data as deafness rates have currently only been extracted for complete deafness [21].

### 2.3. Ethics

No human participants were recruited for this study, and no personal data was reported.

### 2.4. Statistics

The results were reported as percentage uptake with 95% confidence intervals where available. No statistical analysis was applied. The data was discussed as part of a narrative review.

## 3. Results

Results were influenced by the methods used to estimate the number of CI recipients in any given country and the method used to estimate the number of individuals who may meet the criteria for cochlear implantation. These varied both within and across countries according to the method adopted by each paper.

### 3.1. Part 1—Uptake Rates Reported in the Published Literature

Data was extracted from 15 papers. Uptake rates were reported either as the percentage of CI devices or CI recipients, either as a proportion of the population with severe to profound hearing loss (Table 1) or as a proportion of the total population (Table 2).

### 3.2. Part 2—New Uptake Rates for 2019 Based on Global Burden of Disease Report

The Lancet report on global hearing loss provides the estimated number of individuals with hearing loss in that country at a specific time point. The study uses a model of hearing loss based on prevalence data from population-representative surveys in the literature. The total number of CI devices/surgeries currently in a given country up to 2019 was taken from the available literature on the internet. This was divided by the total cases of profound hearing loss per country taken from data reported in the Lancet Global Burden of Disease 2019 report [20], which provided a consistent parameter for the overall population who may benefit from a CI. For our study, we only included those with profound or complete hearing loss as these individuals would comfortably meet the implantation criteria in all countries. No account was taken of other potential criteria such as speech recognition measures. The resulting figures gave a rough estimate for the overall uptake rate in 10 countries in 2019, where overall CI numbers could be identified (Table 3). Data was only available for adults and children combined.

**Figure 2:**
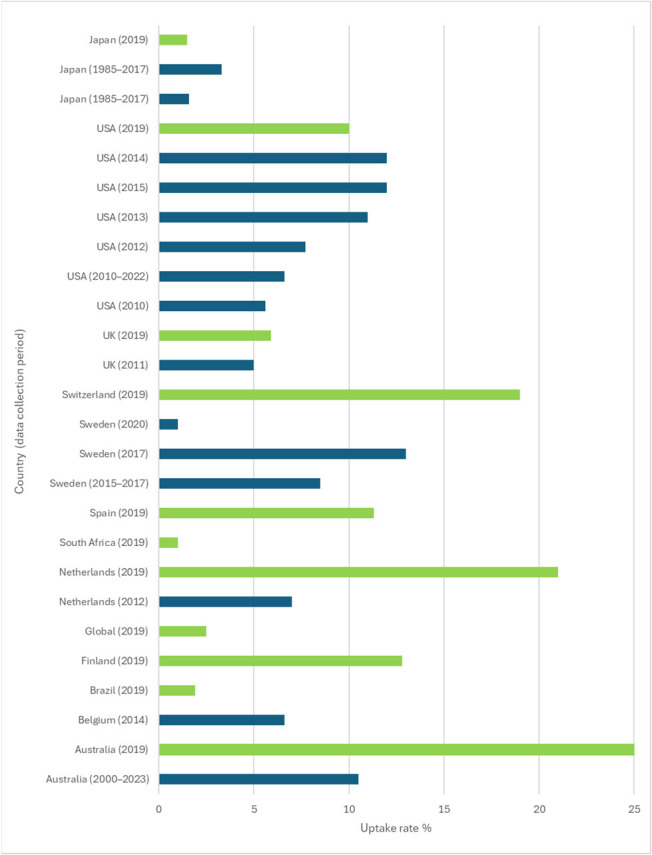
Percentage uptake rates by country for the papers listed in Table 1 and Table 3. Dark blue bars indicate mean uptake rates for data extracted from the literature review and light green bars are new calculations of mean uptake rate based on the 2019 Global Burden of Disease report [8,19,23,24,26,27,28,29,30,31,32].

Combined results from the published data from the systematic review and the new calculations made based on the 2019 Global Burden of Disease report are combined in Figure 2. The highest uptake rates are for Switzerland and the Netherlands (who both have registries) and Australia, where a national database exists. The 2019 calculation represents data for adults and children combined. The implantation rates in children are known to be higher than in adults; thus, these values will be higher than if the figures were for adults only. This can also be seen in the USA data, where adult data reported by Holder et al. (2018) for 2012 is 7.7% and data for adults and children combined reported by Nassiri et al. (2022) for 2013 is 11% [22,23]. It should be noted that different databases for source information were used in these two studies.

### 3.3. Limitations

The numbers of CI recipients in 2019 are estimated for some countries, as data was not available for the exact time frame. However, evidence-based interpolation was applied to make the necessary adjustments, and these are clearly documented. The data sources for Table 3 include “internet searches,” “personal communication” and extrapolations. It was not possible to validate these non-standard sources and therefore there is a high risk of inconsistency in the results. The figures did not account for bilateral implants and reimplantation in all cases. However, cochlear implant reliability is high; thus, this is unlikely to have a large impact on the data, and a failed device is usually replaced with another CI [45]. The impact of bilateral implantation is harder to estimate. It became commonplace to bilaterally implant children from 2010 onwards, and it is estimated that around 30% of CI devices are implanted in bilateral CI patients [23]. The decision to only include profound or complete deafness in the 2019 calculations materially affected uptake estimates and may underestimate the true candidate pool. The numbers of potential CI candidates were based on audiological criteria alone and do not account for any other inclusion criteria such as speech recognition. Some individuals cannot have a CI for medical reasons, while others may decline an assessment if offered. However, these numbers are expected to be small.

## 4. Discussion

The results of the literature review highlight the lack of published data on the uptake rates for cochlear implants, with a notable lack of consensus on the methods used across studies. The number of CI users was not recorded in a consistent way, with studies using the number of surgeries recorded in registries, insurance claim data, the number of devices registered on company databases or the number of individuals followed by the clinics. There was also variation in the methods used to estimate the population of potential CI candidates with severe to profound hearing loss. There is a known lack of reliable epidemiological studies on hearing loss, and the prevalence of severe or greater hearing loss is not well-documented [12]. For example, in the most recent Global Burden of Disease Report (2021), only data for the overall prevalence of hearing loss is reported [46]. In some calculations, prevalence was based on epidemiological studies and in others on numbers from population or audiology databases. The impact of the calculation method used on the uptake rate is highlighted in the variation in uptake rates for Japan (1.5% to 3.3%) and Sweden (1% to 13%) across similar time frames. This means that any assessment of changing uptake rates over time can only be made when the same method is used across multiple years. Nassiri et al. (2022) and Sorkin et al. (2013) both used iData research for the USA and showed that although uptake changed from 6% in 2010 to 11% in 2015, uptake rates barely changed across the next three years, increasing from 11% to 12% [8,23]. De Raeve et al. (2020) also reported little change in the number of implants per 100,000 of population in Europe over a five-year period from 2010 to 2016 [6]. Small increases in the number of implants per head of population for Belgium, Switzerland, Finland and the UK were shown. However, the overall population numbers used do not account for any increased demand over time due to an ageing population or improved neonatal hearing screening programmes.

The overall uptake rates for adults and children combined, calculated for 2019 using the Lancet Global Burden of Disease Study, showed that uptake remains at less than 20% of those with profound hearing loss in most countries. When the global population is considered (including high- to low-income countries), it is merely 2.5%. For our study, we only included those with profound or complete hearing loss, as these individuals would comfortably meet the implantation criteria in all countries, but many of those with severe to profound hearing loss would also be potential candidates [47]. Conversely, a small proportion of individuals with profound hearing loss may not be suitable for a CI due to absent or poorly functioning auditory nerves. The data for the number of CI recipients was sourced where possible from registries but represents what is available from manual searching and is not exhaustive. Where figures are provided by patient groups, their accuracy is unverified. Much of the data reported was for adults and children combined. Adult uptake rates are expected to be considerably lower than paediatric uptake rates [7]. However, the effect of the children in the sample is likely to be smaller than expected as children only represent approximately one third of the cochlear implants sold. We also did not account for more than one device in the same patient, and the figures assume that each device is a unique recipient.

Little published data was available in the literature for Europe beyond that gathered in 2016. Nonetheless, registries are established in Switzerland, Sweden, the Netherlands, France and newly in Germany and the UK [48]. Although data from these registries is not always publicly accessible, they will go some way to addressing the issue of data collection for the number of CI surgeries performed. Access to recent and reliable data on hearing loss is in line with the requirements of the World Health Organisation report on hearing [14]. The intention is that access to this data allows governments to shape policy and ultimately improve funding and increase the supply of CIs. Interestingly, the highest uptake rates were in countries with registries (Switzerland and the Netherlands) or where data is collected nationally (Australia). This may reflect more accurate data collection in these countries or that the registries are already acting to support and promote cochlear implantation as a treatment, possibly at a governmental level. National registries would also reduce the current reliance on industry-funded databases that potentially introduce reporting bias and compromise transparency. Individual country factors also come into play. Japan, for example, is a high-income country with universal government-funded health care where rates of CIs are persistently low. The number of CIs implanted is increasing over time in Japan, but seemingly this is unable to keep up with the numbers of profoundly deaf individuals in their ageing population. As we age, the prevalence of severe to profound deafness increases and although the number of CIs per head of population is increasing in most high-income countries, the rate of growth is not keeping up with the increased demand [12].

There are still considerable barriers to cochlear implantation that have been identified in several studies which reduce the demand for CIs [49,50]. There is poor understanding of hearing loss severity compared to other medical issues and a lack of awareness of the consequences of hearing loss [51]. Treatment is often not sought for many years, and this is exacerbated by the lack of adult hearing screening programmes [52]. There is poor awareness of CIs amongst hearing aid users and a lack of knowledge of referral criteria among professionals [17,53]. Referral pathways are often unclear and complicated, with many hurdles for an individual to negotiate before receiving an implant. Misinformation and worries about surgery may deter individuals from seeking a CI assessment [50]. Although CI users’ support groups can be a powerful tool to guide potential candidate through the process, these are often not accessed until the referral is made. Sociodemographic factors such as age and race are additional barriers to some as well as geographic location, with rural areas often underserved [49,54,55,56]. These barriers are not unique to CIs but also exist for access to hearing aids [57]. However, although the uptake rates for hearing aids overall are only around 20–30%, the uptake increases with hearing loss severity and is around 75% for those with the most severe hearing losses [52,58]. Funding and clinic capacity are both areas to be considered, which undoubtedly place restrictions on the number of implants that can be performed in a country. Nonetheless, waiting lists for adults are non-existent in some areas where uptake is known to be poor (personal communication). This is clearly not the only reason for the low numbers of adults being implanted.

The introduction of mandated National Adult Hearing Screening Programmes and automatic referral triggers would raise awareness of hearing loss and encourage people to take early action [59]. The need for action on hearing loss is pressing and current, with the World Assembly adopting a resolution in February 2025. This highlights the need for hearing care to become an integral component of universal health coverage and integrated into national health plans.

## 5. Conclusions

Global access to cochlear implants for adults and children, averaged across all countries, is extremely low, with only 2.5% of profoundly deaf individuals receiving a CI based on 2019 figures. Despite the cochlear implant awareness activities of recent years, the percentage of profoundly deaf individuals with cochlear implants, even in high-income countries, remains low at ≤20%. Uptake rates are much worse than for hearing aids, particularly in the severe to profound category. Better and more accurate data must be gathered on the number of CI recipients to meet the reporting requirements of the World Health Organisation report on hearing. The uptake figures should be updated using the most recent 2021 Global Burden of Disease report and results from National registries, when available. A better understanding of hearing loss and its consequences in the wider community is needed with improved access to hearing care services, clear clinical guidelines and clear referral pathways for treatment.

## Figures and Tables

**Table 1:** Data extracted from the 12 papers included in the literature review where uptake rates were reported as the percentage of CI devices or CI recipients, as a proportion of the population with severe to profound hearing loss. Studies report adults only or adults and children combined.

Study (Year)	Country	Data Collection Period	CI User Estimation Method	Source CI Candidate Data	Hearing Loss Criteria	Population Group	Uptake Rate (%)
Sorkin (2013) [8]	USA	2010	iData market research report 2010	iData market research report 2010	Severe to profound	Adults and children combined	5.6
Holder (2018) [22]	USA	2012	NIDCD * 2016 data	NIDCD 2004 estimates	Severe to profound	Adults	7.7
Nassiri (2022) [23]	USA	2013	iData market research report 2016	iData market research report 2016	Severe to profound	Adults and children combined	11
		2014					12
		2015					12
De Raeve & van Hardeveld (2013) [24]	Netherlands	2012	Euro-CI users registry	Davis (1995) [25]	>90 dB HL	Adults	7.0
De Raeve (2016) [26]	Belgium	2014	Approved reimbursement	Davis (1995) [25]	>85 dB HL	Adults	6.6
Eikelboom (2025) [19]	Australia	2000–2023	Institute for Health and Welfare surgery data **	Global Burden of Disease	Severe to profound	Adults	10.5
Gumbie (2022) [27]	Sweden	2017	National Otorhinolaryngology registry	National Otorhino-laryngology registry	Severe to profound	Adults	13.0
Kashio (2021) [28]	Japan	1985–2017	National CI registry	Davis (1995) [25]	>90 dB HL	Adults	1.6
				e-Stat registry			3.3
Löfvenberg (2022) [29]	Sweden	2020	National surgical registry	Auditbase audiometry database	≥70 dB HL	Adults	1.0
Raine (2013) [30]	UK	2011	UK CI centre reports	Davis (1995) [25]	Profound	Adults	5.0
Turunen-Taheri (2019) [31]	Sweden	2015–2017	Selected registry sample	Selected registry sample	Severe to profound	Adults	8.5
Zhan (2024) [32]	USA	2010–2022	PearlDiver claims database	PearlDiver claims database	Severe to profound	Adults	6.6

* National Institute of Deafness and Communication Disorders. ** Number of surgeries adjusted for bilateral implants and reimplantation.

**Table 2:** Data extracted from the 3 papers included in the literature review where uptake rates were reported as a proportion of the total population. Studies report adults only or adults and children combined.

Study and Country	Data Collection Period	CI User Estimation Method	Hearing Loss Criteria	Population Group	Number Per 100,000 Population
Nassiri et al. 2023 [33] USA	2015	Prospective registries from two CI manufacturers	Severe to profound	Adults	2.3 *
	2019				3.4 *
Balachandra et al. 2022 [34] USA	2010	Texas Health Care Information Council hospital discharge data	Not defined	Adults and children combined	3.8
	2017				6.9
De Raeve et al. 2020 [6] Multiple countries (listed below)		Publicly available databases	Severe to profound	Adults and children combined	
UK	2010	As above	As above	As above	1.7
	2016	As above	As above	As above	2.2
Switzerland	2010	As above	As above	As above	2.0
	2016	As above	As above	As above	2.7
Sweden	2010	As above	As above	As above	2.3
	2016	As above	As above	As above	2.9
Netherlands	2010	As above	As above	As above	2.7
	2016	As above	As above	As above	3.0
Belgium	2010	As above	As above	As above	1.7
	2016	As above	As above	As above	2.7
Spain	2010	As above	As above	As above	2.0
	2016	As above	As above	As above	1.7
Finland	2010	As above	As above	As above	1.7
	2016	As above	As above	As above	2.7
Germany	2010	As above	As above	As above	4.9
	2016	As above	As above	As above	4.5
Other	2016	As above	As above	As above	≤1

* Measured using number of CIs per 100,000 person years.

**Table 3:** Uptake rate for adults and children combined based on the total numbers implanted by the end of 2019 and estimates of the numbers of individuals with profound and complete deafness from the global report on hearing for 2019 [20]. Where figures were given for a different time frame (usually beyond 2019), information on the annual implantation rates for that country was used to discount the overall number of devices. * Indicates source data is from a validated database or source.

Country and Data Source	Reported CI Numbers	Adjustments Made	Final Number of CI at 2019	Potential CI Candidates Thousands (95% CI)	Uptake Rate % (95% CI)
* Australia Eikelboom et al. (2025) [19] from the National Hospital Morbidity Database	July 2000 to June 2019, *n* = 22,319 adult and child recipients	2000 added from estimates of implants 1990–2000 at 200 per year	24,319	94.9 (75–119)	25 (20–32)
* Brazil Daher et al. (2021) [35] From DATASUS government database	2000–2019, *n* = 10,427 CI surgeries	597 added pre-2000 based on ¼ of the total implantations in Latin America [36]	11,024	547 (432–686)	1.9 (1.6–2.5)
Finland The Finnish Association of Cochlear Implant Recipient Children [37]	Up to 2019, *n *= 1801 individuals		1801	14.0 (11–18)	12.8 (10–16)
* Global National Institute on Deafness and Other Communication Disorders [38]	Up to 2019, *n *= 736,000 registered devices		736,000	30 million (24–36)	2.5 (2–3)
* Japan Kashio et al. (2021) [28]	1985–2017 *n* = 10,778 cases	1222 added based on extrapolation from the graph	12,000	781 (611–985)	1.5 (1.2–1.9)
* Netherlands Independent Platform Cochlear Implantation Foundation (OPCI) [39]	Up to 2023, *n* = 9722 unilateral surgeries	Reduced by 1560 based on number of CI surgeries per year for 2020–2023 from the National CI Registry	8162	39.4 (30–50)	21 (16–27)
South Africa Bhamjee et al. (2022) [40]	Up to 2019, *n *= 2877 individuals		2877	278 (219–350)	1 (0.8–1.3)
Spain Associations of Cochlear Implant Patients of Spain [41]	Up to 2024, *n* = 23,000 individuals	23,000 discounted at 3000 per year for 2023 and 2022, and 1500 per year for 2021 and 2020 based on estimates from market data [42]	14,000 (discounted at 3000 CIs per year)	123.9 (97–157)	11.3 (14.4–8.9)
* Switzerland Swiss Cochlear Implant Register [43]	Up to 2019, *n *= 3882 surgeries		3882	19.8 (15–25)	19.6 (11.5–26)
* UK British Cochlear Implant Group [44]	Up to 2019, *n *= 18,000 total CIs maintained		18,000	303 (242–375)	5.9 (4.8–7.4)
* USA National Institute on Deafness and Other Communication Disorders [38]	Up to 2019, *n* = 183,100 registered devices		183,100	1800 (1429–2250)	10 (8–13)

## Data Availability

Dataset available on request from the authors.
